# 755. Predictors of Mortality in Invasive Mold Infections Among Patients with Hematologic Malignancies and Hematopoietic Stem Cell Transplant Recipients

**DOI:** 10.1093/ofid/ofac492.040

**Published:** 2022-12-15

**Authors:** Aanchal Gupta, Nischal Ranganath, Maria A Mendoza, Infectious Diseases, Sharon Deml, Nancy Wengenack, Paschalis Vergidis

**Affiliations:** UMass Chan Medical School, Natick, MA; Mayo Clinic College of Medicine and Science, Rochester, Minnesota; Mayo Clinic Rochester, Rochester, Minnesota; Mayo Clinic, Rochester, Minnesota; Mayo Clinic, Rochester, Minnesota; Mayo Clinic, Rochester, Minnesota

## Abstract

**Background:**

Invasive Mold Infections (IMI) cause significant morbidity and mortality, particularly in patients with hematologic malignancies (HM) and hematopoietic stem cell transplant (HSCT) recipients. Although the risk has significantly decreased with the use of triazole agents as primary prophylaxis, breakthrough infections still occur. Risk factors for IMI have been well-described. Limited data exist on factors predicting mortality.

**Methods:**

We identified cases using the Cancer registry and Microbiology data at Mayo Clinic Minnesota from 11/19/2011–8/6/2021 and Mayo Clinic Arizona and Florida from 09/13/2017–11/19/2021. We conducted a retrospective review to identify cases of proven/probable IMI per EORTC/MSG criteria. Patients who received at least 7 days of anti-mold prophylaxis were considered having breakthrough infection (Br-IMI). Survival function was estimated using the Kaplan-Meier method. We evaluated predictors of mortality using Cox regression analysis.

**Results:**

401 cases of IMI were included (80.2% aspergillosis, 10.2% fusariosis, 8.4% mucormycosis, 1.2% scedosporiosis). 189 patients had proven/probable and 212 had possible disease. 12-week mortality for the entire cohort was 33%. There were 87 cases of Br-IMI (21.6%) on mold-active prophylaxis (posaconazole n=54, voriconazole n=26, isavuconazole n=5, itraconazole n=1, liposomal amphotericin B n=1).

A comparison between patients not receiving prophylaxis and those with Br-IMI is shown in Table 1. Higher proportion of patients with Br-IMI had prolonged neutropenia/lymphopenia and graft-versus-host disease involving the gastrointestinal tract or lungs. Positive *Aspergillus* galactomannan (index ≥ 1.0) was seen more in patients who were not on mold-active prophylaxis. Mortality was 31.5% in patients not receiving prophylaxis and 41.3% with Br-IMI (Figure 1). In multivariate analysis, age, prolonged neutropenia, active HM, Br-IMI and HSCT were associated with increased mortality (Table 2).

**Conclusion:**

In this cohort, the rate of Br-IMI was significant despite the use of primary prophylaxis (mainly posaconazole). Mortality was higher in patients with Br-IMI compared to those not receiving mold-active prophylaxis. Therefore, clinicians should remain alert to the possibility of Br-IMI.

**Disclosures:**

**Paschalis Vergidis, MD**, AbbVie: DSMB|Cidara: Grant/Research Support|Scynexis: Grant/Research Support.

